# *Helicobacter suis* induces changes in gastric inflammation and acid secretion markers in pigs of different ages

**DOI:** 10.1186/s13567-017-0441-6

**Published:** 2017-06-15

**Authors:** C. De Witte, B. Devriendt, B. Flahou, I. Bosschem, R. Ducatelle, A. Smet, F. Haesebrouck

**Affiliations:** 1 Department of Pathology, Bacteriology and Avian Diseases, Faculty of Veterinary Medicine, Ghent University, Merelbeke, Belgium; 2 Department of Virology, Parasitology, Immunology, Faculty of Veterinary Medicine, Ghent University, Merelbeke, Belgium; 3 Laboratory of Experimental Medicine and Pediatrics, Faculty of Medicine and Health Sciences, Antwerp University, Antwerp, Belgium

## Abstract

Gastric mRNA expression of markers for acid secretion and inflammation and presence of gastric ulceration was studied in naturally *Helicobacter suis*-infected and non-infected 2–3 months old, 6–8 months old and adult pigs. In *H. suis*-infected 2–3 months old pigs, IL-8 and IL-1β transcript levels were upregulated in the pyloric gland zone, indicating an innate immune response. A similar response was demonstrated in the fundic gland zone of adult pigs, potentially due to a shift of *H. suis* colonization from the pyloric to the fundic gland zone. A Treg response in combination with decreased expressions of IL-8, IL-17A and IFN-γ was indicated to be present in the *H. suis*-infected 6–8 months old pigs, which may have contributed to persistence of *H. suis*. In *H. suis*-infected adult pigs, a Treg response accompanied by a Th17 response was indicated, which may have played a role in the decreased number of *H. suis* bacteria in the stomach of this age group. The decreased G-cell mass and upregulated expression of somatostatin indicated decreased acid secretion in *H. suis*-infected 6–8 months old pigs. In *H. suis*-infected adult pigs, upregulation of most markers for gastric acid secretion and increased G-cell mass was detected. Presence of severe hyperkeratosis and erosions in the non-glandular part of the stomach were mainly seen in the *H. suis*-positive groups. These results show that *H. suis* infection affects the expression of markers for acid secretion and inflammation and indicate that these effects differ depending on the infection phase.

**Electronic supplementary material:**

The online version of this article (doi:10.1186/s13567-017-0441-6) contains supplementary material, which is available to authorized users.

## Introduction

Gastric ulceration is a common disease entity of pigs worldwide, with prevalences of up to 93% [1]. Although the disease outcome is often subclinical, animal welfare issues as well as economic losses due to decreased daily weight gain, decreased feed intake and sudden death, are of major importance [1]. The etiology seems to be multifactorial. Indeed, several factors, including diet particle size [2], management [3], gastric microbiota composition, infection with *Helicobacter suis* [4, 5], and hormonal changes [6] have been hypothesized to be involved. The pathogenesis of porcine gastric ulceration, however, remains largely unknown [1]. What we do know is that, in contrast to other animal species, in pigs gastric ulcers develop almost exclusively in the *Pars œsophagea*, a small area around the opening of the œsophagus which does not contain glands. Since this stomach region is not protected by mucus, it is highly susceptible to irritation with for instance hydrochloric acid, produced in the fundic gland zone of the porcine stomach [1]. Chronic insult of the *Pars œsophagea* results in hyperkeratosis, erosion and finally ulceration.

Pigs are commonly infected with the zoonotic pathogen *H. suis* [1]. This pathogen mainly colonizes the fundic and pyloric gland zone of the porcine stomach, inducing inflammation and a decreased daily weight gain [5]. It has been hypothesized that alterations in hydrochloric acid production in the glandular region of the stomach, associated with chronic *H. suis* infections, may play a role in the pathogenesis of swine gastric ulceration [1, 4, 5]. Hellemans et al. [4] demonstrated a tropism of *H. suis* for the gastric acid producing parietal cells. Histological analysis of the stomach of *H. suis*-infected pigs at slaughter age, has revealed that these bacteria are often found in close vicinity of parietal cells and even inside the canaliculi of these cells [4]. In addition, *H. suis* can cause degenerative changes and necrosis of parietal cells in porcine, human and rodent models of gastric disease [7, 8]. Recent reports indicated that *H. suis* may disturb homeostasis of porcine parietal cells and affect their expression of genes encoding H+/K+ ATPase [9]. The latter is an enzyme typically associated with parietal cells and is involved in gastric acid production by these cells. Not only parietal cells, but also gastrin producing G-cells and somatostatin producing D-cells can be altered during *H. suis* infection [10]. Gastrin stimulates and somatostatin suppresses gastric acid production through their association with CCK-B and SST2 receptors on parietal cells, respectively, suggesting that *H. suis* infection may indeed affect gastric acid secretion through different mechanisms.

The main objectives of this study were to obtain further insights in the mechanisms involved in persistence of *H. suis* in the porcine stomach and in its effects on gastric acid secretion and lesion development. This was studied in naturally *H. suis* infected pigs during the acute and chronic phases of infection. Therefore, the mRNA expression of different cytokines, chemokines and markers for gastric acid secretion was studied, the parietal cell, D-cell and G-cell mass was analyzed and the severity of *Pars œsophageal* lesions was determined in *H. suis* infected and non-infected 2–3 months old pigs, 6–8 months old pigs and adult sows.

## Materials and methods

### Sampling of porcine stomachs

Sixty-eight stomachs of 6–8 months old pigs and 60 stomachs of adult sows (1–3 years old) were collected over a period of 10 months from 2 slaughterhouses in Flanders, Belgium. The pigs originated from different herds. The stomachs of the 6–8 months old pigs had also been used in a previous study [11]. In addition, stomachs of 34, 2–3 months old pigs were collected from 2 different pig herds (17 samples from each herd). The stomachs were transported immediately to the laboratory and stored at 4 °C until further examination within 2 h. The stomachs were opened along the greater curvature and rinsed with sterile tap water. Based on the method of Hessing [12], mucosal lesions of the *Pars œsophagea* were scored as follows: score 0 for normal mucosa, score 1 for mild hyperkeratosis covering less than 50% of the surface, score 2 for severe hyperkeratosis covering more than 50% of the surface, score 3 for hyperkeratosis with few erosions, score 4 for hyperkeratosis with several erosions and score 5 for hyperkeratosis with many erosions or ulceration. Using autoclaved tweezers and scalpels, biopsies of 40–50 mg consisting of mucosa and submucosa were taken from the *Pars œsophagea* as well as from the cardiac, fundic and pyloric gland zone for quantification of *H. suis* DNA by qPCR. In addition, biopsies consisting of mucosa and submucosa were taken from the fundic and pyloric gland zones to determine mRNA expression levels of genes encoding host factors (markers) involved in gastric acid secretion and inflammation. In order to correlate altered markers with gastritis and the number of parietal cells, D-cells and G-cells, biopsies consisting of mucosa, submucosa and tunica muscularis were taken from fundic and pyloric gland zones, fixed in 10% phosphate-buffered formalin and used for histopathology and immunohistochemistry.

### *H. suis* quantification

DNA was extracted from the biopsies of each stomach region, using the Isolate II Genomic DNA Kit^®^ (Bioline, Taunton, USA), according to the instructions of the manufacturer. The presence of *H. suis* DNA was determined using a species-specific, real time quantitative (RT)-PCR based on the *ureA* gene [13]. The copy number of the obtained amplicons was calculated and converted to the number of *H. suis* bacteria per mg gastric tissue, by including tenfold dilutions of an external standard consisting of a 1236 bp segment of the *ureAB* gene cluster from *H. suis* strain HS5 [14].

### Histopathology and immunohistochemistry

The biopsies were embedded in paraffin, sectioned at 5 μm, rehydrated, deparaffinized, stained with hæmatoxylin and eosin, dehydrated and finally mounted with a coverslip for light microscopic evaluation. The severity of gastritis was scored according to the Updated Sydney System with some modifications [5, 15]. Both diffuse infiltration with inflammatory cells and the presence of lymphoid aggregates and lymphoid follicles in the mucosa and submucosa were taken into consideration. The infiltration of mononuclear and polymorphonuclear cells was scored as follows: score 0 for absence of infiltration, score 1 for mild infiltration, score 2 for moderate infiltration and score 3 for marked infiltration. In addition, the formation of lymphoid follicle formation was scored as follows: score 0 for absence of lymphoid aggregates, score 1 for presence of a small number of lymphoid aggregates (*n* < 5), score 2 for a large number of lymphoid aggregates (*n* ≥ 5) and/or the presence of 1 organized lymphoid follicle and score 3 for the presence of at least 2 organized lymphoid follicles. Based on the scoring of the diffuse infiltration with inflammatory cells and the presence of lymphoid aggregates and lymphoid follicles, an overall gastritis score was obtained. Therefore, the average score was calculated for each *H. suis*-negative and *H. suis*-positive age group and this for the pyloric and fundic gland zone. When an overall score of 0 ≤ *n* ≤ 1; 1 < *n* ≤ 2 or 2 < *n* ≤ 3 was obtained, the gastritis was considered as mild, moderate and severe, respectively.

To determine the number of parietal cells, D-cells and G-cells, 3 consecutive sections of 5 μm were cut from the paraffin embedded tissues. After rehydration and deparaffinization, heat-induced antigen retrieval was performed in citrate buffer (pH 6.0) using a microwave oven. Slides were incubated with 3% H_2_O_2_ in methanol (5 min) to block endogenous peroxidase activity and with 30% goat serum (30 min) to block non-specific reactions. Parietal cells were identified by immunohistochemical staining for the H+/K+ ATPase using a mouse monoclonal antibody (1/200; Abcam Ltd, Cambridge, United Kingdom) and a biotinylated goat anti-mouse IgG antibody (1/200; Agilent Technologies, Santa Clara, California, USA) [9]. D-cells and G-cells were identified by immunohistochemical staining using a rabbit polyclonal anti-somatostatin and anti-gastrin antibody, respectively (1/600; Agilent Technologies, Santa Clara, California, USA) and a biotinylated goat anti-rabbit IgG antibody (1/600; Agilent Technologies, Santa Clara, California, USA). After rinsing, the sections were incubated with a streptavidin–biotin-HRP complex (Agilent Technologies, Santa Clara, California, USA) [10]. The color was developed with diaminobenzidine tetrahydrochloride (DAB) and H_2_O_2_. Finally, positive D-cells and G-cells were counted in five randomly chosen high power fields (magnification: ×400), both in the fundic and pyloric gland zone. The average number of positive cells per high power field was then calculated for each pig in both stomach regions. As a positive control for the parietal cell staining, the fundic gland zone of a non-*H. suis* infected pig was used, as this zone is known to contain large numbers of these cells [10]. The pyloric gland zone of this pig was used as a positive control for D-cells and G-cells staining. This zone indeed contains large numbers of these cell types [10]. Negative controls to confirm the specificity of the secondary antibodies were obtained by incubating the sections without the primary antibodies. In addition, the cardiac gland zone was also used as a negative control, as this stomach region is known to contain only mucus and bicarbonate producing cells [10].

### Expression analysis of markers for inflammation and gastric acid secretion

RNA was extracted from the gastric biopsies using the RNeasy Mini Kit^®^ (Qiagen, Hilden, Germany) according to the manufacturer’s instructions. The obtained RNA concentrations were measured using a NanoDrop^®^ spectrophotometer (Isogen Life Science, Utrecht, The Netherlands), after which the concentration of all samples was adjusted to 1 μg/μL, followed by cDNA synthesis using the iScript™ cDNA Synthesis Kit (Bio-Rad, Hercules, California, USA). Expression of genes encoding host factors involved in gastric acid secretion (H+/K+ ATPase, Sonic Hedgehog, KCNQ1, gastrin, the cholinergic muscarinic M3 receptor, somatostatin, the histamine H2 receptor and the gastrin CCK-B receptor), mucosal integrity (claudin 18) and inflammation (IL-4, IL-8, IL-10, IL-17A, IL-1β, IFN-γ and CXCL13) was analyzed. HPRT, Cyc5 and ACTB have been shown to have a stable mRNA expression and were therefore included as reference genes [9]. All primer sequences are shown in the Additional file 1. The mRNA expression levels of the reference and target genes were quantified using RT-qPCR, as described earlier [16]. No-template control reaction mixtures were included and all samples were run in duplicate. The threshold cycle (Ct)-values were first normalized to the geometric mean of the Ct-values of the reference genes. Fold changes were calculated using ΔΔCT method with the means of Ct-values from the *H. suis* negative pigs. Finally, for each target gene, the results were expressed as fold changes of the mRNA expression of *H. suis* positive pigs relative to mRNA expression levels of *H. suis* negative pigs and this for each age group separately.

### Statistical analysis

Statistical analysis was performed using SPSS statistics 24^®^ (IBM, New York, USA). Differences in severity of *Pars œsophageal* lesions, severity of gastritis, number of parietal cells, D-cells and G-cells and fold changes of the markers for gastric acid secretion and inflammation between the *H. suis*-negative and *H. suis*-positive groups were investigated using the non-parametric Kruskal-Wallis H test with Bonferroni correction. A *p* value ≤ 0.05 was considered to be significant. Correlations between mucosal lesions, severity of gastritis, number of parietal cells, D-cells and G-cells, fold changes and the number of *H. suis* bacteria were examined using the Pearson correlation coefficient. Differences were considered statistically significant at *p* ≤ 0.05.

## Results

### *H. suis* prevalence and association with mucosal lesions, gastritis and number of parietal cells, D-cells and G-cells

#### Two–3 months old pigs

**Figure 1:**
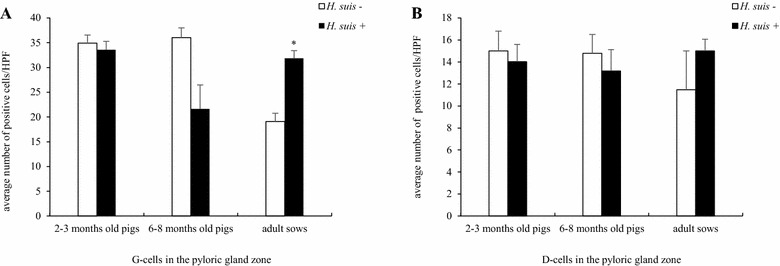
**A, B The number of G-cells (A) and D-cells (B) present in the pyloric gland zone of** ***H. suis*** **-negative (−) and** ***H. suis*** **-positive (+) pigs of different ages.** Data are shown as the average number of G-positive and D-positive cells of each age group with standard deviation. Statistical differences were calculated using the non-parametric Kruskal–Wallis H test. * Significant differences between the *H. suis*-negative and *H. suis*-positive pigs (*p* < 0.01); HPF, high power field.

The prevalence of *H. suis* was 47% and the average number of *H. suis* bacteria per mg tissue was higher in the pyloric gland zone than in the other stomach regions (*p* < 0.001) (Additional file 2). Almost all pigs had *H. suis* DNA in the fundic and pyloric gland zone (=15/16). On gross examination, all pigs showed an intact mucosa or mild hyperkeratosis of the *Pars œsophagea* (Table 1), with moderate gastritis in the fundic and pyloric gland zone (Additional file 3). The scores for *Pars œsophageal* lesions and gastritis were not significantly different between the *H. suis*-negative and *H. suis*-positive pigs. Similarly, the number of G-cells and D-cells in the pyloric gland zone did not differ between the *H. suis*-negative and *H. suis*-positive group (Figures 1A and B). A small number of G-cells and D-cells was detected in the fundic gland zone of the pigs, varying from 0 to 3 per high power field and independent from the *H. suis* status (data not shown). In the fundic gland zone of both *H. suis*-infected and non-infected pigs, the number of parietal cells was high in each high power field (> 800/field), making the counting impossible. A small number of parietal cells was detected in the pyloric gland zone of the pigs, varying from 0 to 2 per high power field and independent from the *H. suis* status (data not shown). For the *H. suis*-positive pigs, statistical analysis did not reveal significant correlations between mucosal lesions, gastritis and the number of *H. suis* bacteria. Analysis of gene expression, histopathology and immunohistochemistry was done on samples from all pigs in this age category (see below).

**Table 1:** **General overview of the score distribution of lesions (%) in the** ***Pars œsophagea*** **of pigs of different ages**

Age group	*Pars œsophagea*—lesion score					
	0 (%)	1 (%)	2 (%)	3 (%)	4 (%)	5 (%)
2–3 months old (*n* = 34)	38	44	18	0	0	0
* H. suis* positive (*n* = 16)	37	44	19	0	0	0
* H. suis* negative (*n* = 18)	39	44	17	0	0	0
6–8 months old (*n* = 68)	2	27	50	13	3	5
* H. suis* positive (*n* = 55)	0	13	61	17	4	6
* H. suis* negative (*n* = 13)	8	92	0	0	0	0
Adult sows (*n* = 60)	0	5	20	15	10	50
* H. suis* positive (*n* = 55)	0	5	20	13	9	53
* H. suis* negative (*n* = 5)	0	0	20	40	20	20

Score 0 = normal mucosa, 1 = mild hyperkeratosis covering less than 50% of the surface, 2 = severe hyperkeratosis covering more than 50% of the surface, 3 = hyperkeratosis with few erosions, 4 = hyperkeratosis with several erosions, 5 = hyperkeratosis with many erosions or ulceration, *n* = total number of investigated pigs’ stomachs per age group. The data are shown as the percentage of pigs showing a certain lesion score.

#### Six–8 months old pigs

*Helicobacter suis* was detected in 81% of the investigated stomachs. The average number of *H. suis* bacteria per mg tissue was similar for the fundic and pyloric gland zone (Additional file 2). *H. suis* DNA was detected in the fundic and pyloric gland zone of all *H. suis*-infected pigs. Severe hyperkeratosis and erosions were only seen in the *H. suis*-positive group (Table 1). The number of D-cells did not differ between the *H. suis*-negative and *H. suis*-positive group, whereas the number of G-cells was decreased in the *H. suis*-positive group (*p* = 0.054) (Figures 1A and B). A small number of G-cells and D-cells was detected in the fundic gland zone of the pigs, varying from 0 to 3 per high power field and independent from the *H. suis* status (data not shown). In the fundic gland zone of both *H. suis*-infected and non-infected pigs, the number of parietal cells was high in each high power field (> 800/field), making the counting impossible. A small number of parietal cells was detected in the pyloric gland zone of the pigs, varying from 0 to 2 per high power field and independent from the *H. suis* status (data not shown). No significant correlations were detected between the number of *H. suis* bacteria and severity of gastritis. All *H. suis*-negative pigs (*n* = 13), 5 pigs with > 1000 *H. suis* bacteria/mg tissue in the fundic gland zone, 5 pigs with > 1000 *H. suis* bacteria/mg tissue in the pyloric gland zone and 5 pigs with < 100 *H. suis* bacteria/mg tissue in both the fundic and pyloric gland zone were selected for analysis of gene expression, histopathology and immunohistochemistry.

#### Adult sows

*Helicobacter suis* was detected in the stomach of 55/60 sows (92%). In contrast with the other age groups, the average number of *H. suis* bacteria per mg tissue was higher in the fundic gland zone than in the other stomach regions (*p* < 0.01) (Additional file 2). All *H. suis*-infected sows had *H. suis* DNA in the fundic and pyloric gland zone. Ulceration was mainly found in the *H. suis*-positive sows, although this was not significantly different from the *H. suis*-negative group (Table 1). No significant differences were detected in the severity of gastritis between the *H. suis*-negative and *H. suis*-positive pigs (Additional file 3). A significant positive correlation was found, however, between the number of *H. suis* bacteria per mg gastric tissue and lymphoid infiltration in the fundic gland zone (*p* < 0.001). The number of D-cells did not differ between the *H. suis*-negative and *H. suis*-positive group, while the number of G-cells was increased in the *H. suis*-positive group (*p* = 0.002) (Figures 1A and B; Additional file 4). A small number of G-cells and D-cells was detected in the fundic gland zone of the pigs, varying from 0 to 3 per high power field and independent from the *H. suis* status (data not shown). In the fundic gland zone of both *H. suis*-infected and non-infected pigs, the number of parietal cells was high in each high power field (> 800/field), making the counting impossible. A small number of parietal cells was detected in the pyloric gland zone of the pigs, varying from 0 to 2 per high power field and independent from the *H. suis* status (data not shown). All *H. suis*-negative sows (*n* = 5) were selected for gene expression, histopathological and immunohistochemical analysis. From the *H. suis*-positive group, these analyses were performed on 20 stomachs, selected as follows: 5 sows with > 1000 *H. suis* bacteria/mg tissue in both the fundic and pyloric gland zone, 5 sows with > 1000 *H. suis* bacteria/mg tissue in the fundic gland zone, 5 sows with > 1000 *H. suis* bacteria/mg tissue in the pyloric gland zone and 5 sows with < 100 *H. suis* bacteria/mg tissue in both the fundic and pyloric gland zone.

#### Comparison of the different age groups

**Figure 2:**
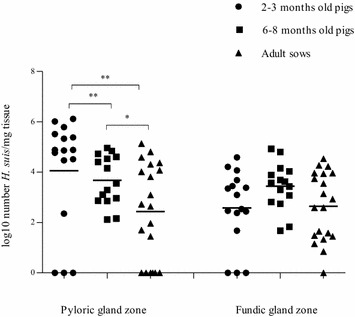
**Comparison of the number of** ***H. suis*** **bacteria in the fundic and pyloric gland zone of pigs of different ages.** Data are shown as log10 values of the number *H. suis* bacteria per mg tissue. Pigs in which no *H. suis* infection was detected, were set as 0. Individual pigs are shown as figures around the mean (lines). Statistical differences were calculated using the non-parametric Kruskal–Wallis H test. *p < 0.05; **p < 0.001 significant differences between the *H. suis*-positive age groups.

The scores given for *Pars œsophageal* lesions were significantly different between each age group (*p* < 0.001), with more severe lesions in adult sows, followed by 6–8 months old pigs. In contrast, the scores for lymphoid infiltration and lymphoid follicle formation did not differ significantly between the age groups, nor did the number of parietal cells, G-cells and D-cells (Figures 1A and B; Additional file 3). Although the prevalence of *H. suis* progressively increased with age, the number of *H. suis* bacteria per mg tissue decreased with age, especially in the pyloric gland zone (*p* < 0.05; Figure 2). Significantly higher scores for lymphoid infiltration and lymphoid follicle formation were found in the pyloric gland zone compared to the fundic gland zone (*p* < 0.005), independent from the *H. suis* status, and this in all age groups.

### Gene expression analysis of markers for inflammation

#### Two–3 months old pigs

**Figure 3:**
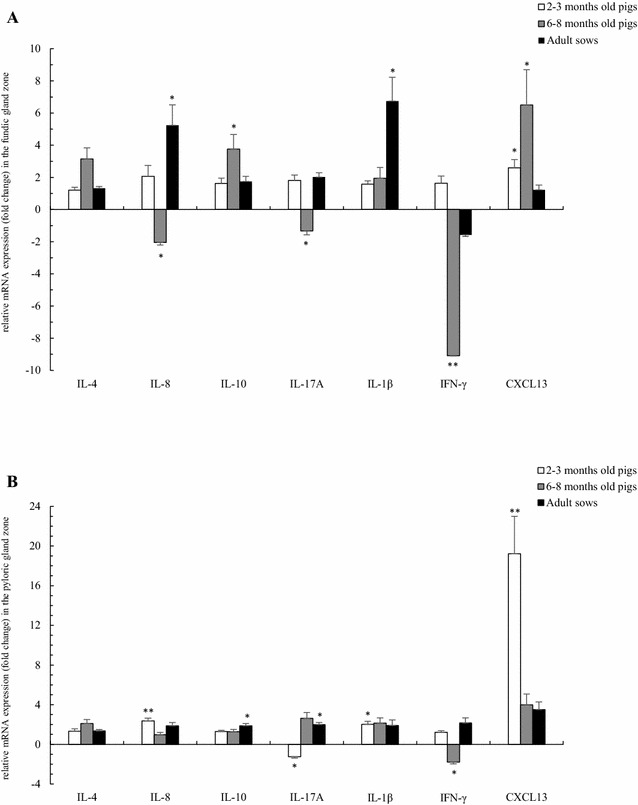
**A, B General overview of gene expression analysis of markers for inflammation in the fundic (A) and pyloric (B) gland zone of** ***H. suis*** **-infected pigs of different ages.** The data are presented as fold changes in gene expression normalized to 3 reference genes and relative to a *H. suis*-negative control group which is considered as 1. The fold changes are shown as means with the standard error of the mean. Statistical differences were calculated using the non-parametric Kruskal–Wallis H test. *p < 0.05; **p < 0.001 significant differences between the *H. suis*-positive pigs and *H. suis*-negative pigs.

Compared to the non-infected group, the mRNA expression of CXCL13 was significantly upregulated in the fundic and pyloric gland zones of the *H. suis*-infected group (*p* = 0.027 and < 0.001, respectively), as well as the IL-8 and IL-1β transcript levels in the pyloric gland zone (*p* = 0.001 and 0.034, respectively). In contrast, IL-17A was significantly downregulated in the pyloric gland zone (*p* = 0.039) (Figures 3A and B; Additional file 5). Since significant correlations were found between both, the altered fold changes of IL-8, IL-17A, IL-1β and CXCL13 were more pronounced in pigs with a high number of *H. suis* bacteria per mg gastric tissue (Additional file 6).

#### Six–8 months old pigs

Upregulated expression of inflammatory cytokines was as described in the study of Bosschem et al. [11]. In brief, compared to the non-infected group, in the *H. suis*-positive group, significant upregulations of IL-10 and CXCL13 were detected in the fundic gland zone (*p* = 0.047 and 0.011, respectively). The expressions of IL-4 in the fundic gland zone and that of IL-4, IL-17A and CXCL13 in the pyloric gland zone were also upregulated, although not significantly. A significant downregulation of IL-8 and IL-17A was detected in the fundic gland zone (*p* = 0.040 and 0.029, respectively), while IFN-γ was significantly downregulated in both fundic and pyloric gland zone (*p* < 0.001 and = 0.005, respectively) (Figures 3A and B; Additional file 5). Since significant correlations were found between both, the altered fold changes of IL-4, IL-8, IL-10, IL-17A, IFN-γ and CXCL13 were more pronounced in pigs with > 1000 *H. suis* bacteria/mg gastric tissue (Additional file 6).

#### Adult sows

Compared to the non-infected sows, in the *H. suis*-infected adult sows, the mRNA expression of IL-8 and IL-1β was significantly upregulated in the fundic gland zone (*p* = 0.018 for IL-8 and 0.037 for IL-1β), while IL-10 and IL-17A were upregulated in the pyloric gland zone of *H. suis*-infected sows (*p* = 0.019 and 0.042, respectively). Although not significantly, increased IL-10 and IL-17A mRNA expression was also detected in the fundic gland zone and in the pyloric gland zone for IFN-γ and CXCL13 (Figures 3A and B; Additional file 5). Since significant correlations were found between both, the altered fold changes of IL-8, IL-10, IL-17A, IL-1β, IFN-γ and CXCL13 were more distinct in pigs with > 1000 *H. suis* bacteria/mg gastric tissue (Additional file 6).

### Gene expression analysis of markers for gastric acid secretion

#### Two–3 months old pigs

**Figure 4:**
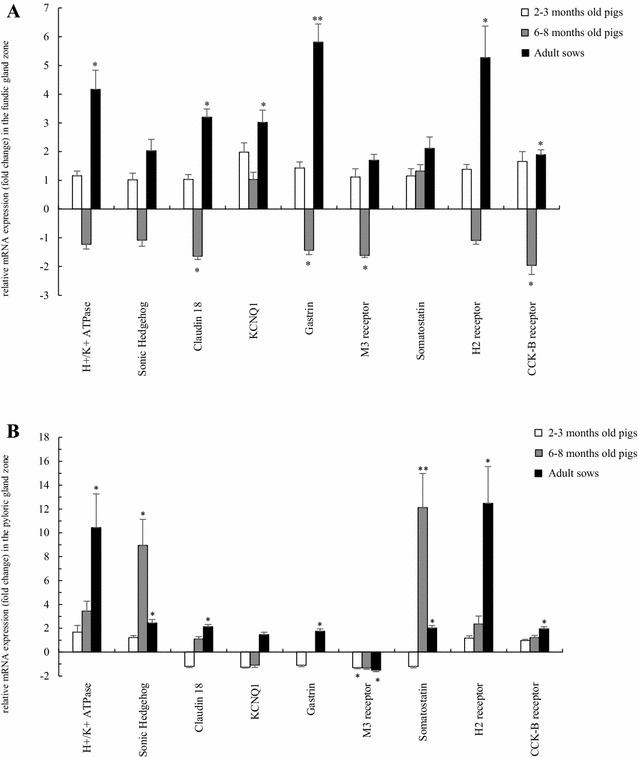
**A, B General overview of gene expression analysis of markers for gastric acid secretion in the fundic (A) and pyloric (B) gland zone of** ***H. suis*** **-infected pigs of different ages.** The data are presented as fold changes in gene expression normalized to 3 reference genes and relative to a *H. suis*-negative control group which is considered as 1. The fold changes are shown as means with the standard error of the mean. The average fold change of gastrin in the pyloric gland zone of 6–8 months old pigs is not shown, since these values were too high (231.97 ± 64.63). Statistical differences were calculated using the non-parametric Kruskal–Wallis H test. *p < 0.05; **p < 0.001 significant differences between the *H. suis*-positive pigs and *H. suis*-negative pigs.

Compared to the non-infected group, in the *H. suis*-infected 2–3 months old pigs, the majority of the markers for gastric acid secretion were not altered, except for a significant downregulated expression of the M3-receptor in the pyloric gland zone (*p* = 0.027). The expression of KCNQ1 was upregulated in the fundic gland zone, while somatostatin was downregulated in the pyloric gland zone, although not significantly (Figures 4A and B; Additional file 7). Since significant correlations were found between both, the altered fold changes of KCNQ1, M3-receptor and somatostatin were more pronounced in pigs with > 1000 *H. suis* bacteria/mg per mg gastric tissue (Additional file 8). In addition, since significant correlations were found between both markers, the altered fold change of somatostatin was more pronounced in pigs with a high expression of CXCL13 and IL-1β (Additional file 9).

#### Six–8 months old pigs

Compared to the non-infected group, in the *H. suis*-infected group claudin 18, gastrin, M3 receptor and CCK-B receptor were significantly downregulated in the fundic gland zone (*p* = 0.022, 0.040, 0.002 and 0.004, respectively), whereas Sonic Hedgehog and somatostatin mRNA levels were significantly upregulated in the pyloric gland zone (*p* = 0.048 and 0.007, respectively). The H+/K+ ATPase expression in the pyloric gland zone was upregulated as well, although not significantly. In the pyloric gland zone of 9 *H. suis*-infected pigs, the expression of gastrin was upregulated (Figures 4A and B; Additional file 7), while for 6 *H. suis*-infected pigs the expression was not altered. Since significant correlations were found between both, the altered fold changes of H+/K+ ATPase, Sonic Hedgehog, claudin 18, gastrin, M3 receptor, somatostatin and CCK-B receptor were more pronounced in pigs with > 1000 *H. suis* bacteria/mg gastric tissue (Additional file 8). In addition, since significant correlations were found between both markers, the altered fold changes of claudin 18, M3 receptor, somatostatin and CCK-B receptor were more pronounced in pigs with lower expressions of IL-8, IL-17A and IFN-γ (Additional file 9).

#### Adult sows

Compared to the non-infected sows, most markers for gastric acid secretion were upregulated in the *H. suis*-infected sows. The expression of genes encoding H+/K+ ATPase, claudin 18, H2 receptor and CCK-B receptor were significantly upregulated in the fundic and pyloric gland zone of the *H. suis*-infected sows (*p* = 0.049, 0.002, 0.019, 0.049, 0.002, 0.012, 0.015 and 0.012, respectively). In addition, KCNQ1 and gastrin transcript levels were significantly upregulated in the fundic gland zone (*p* = 0.012 and < 0.001, respectively), whereas a significant downregulated mRNA expression of the M3 receptor in the pyloric gland zone was noticed (*p* = 0.049). An upregulated expression of Sonic Hedgehog was also detected in the fundic and pyloric gland zone, although not significant (Figures 4A and B; Additional file 7). Since significant correlations were found between both, the altered fold changes of genes encoding H+/K+ ATPase, Sonic Hedgehog, claudin 18, KCNQ1 and CCK-B receptor were more pronounced in pigs with > 1000 *H. suis* bacteria/mg gastric tissue (Additional file 8). In addition, since significant correlations were found between both markers, the altered fold changes of genes encoding H+/K+ ATPase, Sonic Hedgehog, claudin 18, gastrin and CCK-B receptor were more pronounced in pigs with high expressions of IL-10, IL-17A and IFN-γ (Additional file 9). Furthermore, since significant positive correlations were found between both, an increased G-cell number may have contributed to the increased expression of gastrin in both fundic and pyloric gland zone (*r* = 0.562, *p* = 0.003; *r* = 0.465, *p* = 0.022, respectively).

## Discussion

In the present study, the prevalence of *H. suis* was 47% in 2–3 months old pigs and increased to 81% in pigs at slaughter age, which is in line with the results of previous studies [17–19].

The prevalence of *H. suis* was very high in adult animals, indicating that the host immune response is not able to clear the infection. In a recent study, Bosschem et al. [11] showed that *H. suis* induces a semimaturation of porcine monocyte-derived dendritic cells, characterized by increased expression of CD25, CD80/86 and CD40, but impaired expression of MHC class II molecules on the surface of these cells. It was suggested that this impaired dendritic cell response may elicit the expansion of Treg cells, which may help to establish a chronic infection as Treg cells are immune-suppressive and tolerogenic [20]. Indeed, a tolerogenic immune response was indicated to be present in this study, since the Treg cell-associated cytokine IL-10 was upregulated in both *H. suis*-infected 6–8 months old pigs and adult sows. In addition, the downregulated expressions of IL-8, IL-17A and especially IFN-γ indicated presence of an immune-suppressive environment in the *H. suis*-infected 6–8 months old pigs, which may have contributed to the establishment of a chronic infection. In the *H. suis*-infected adult sows, however, the mRNA expression of the Th17 cell-associated IL-17A was upregulated. We also found a shift in colonization of *H. suis* from the pyloric gland zone during the more acute phase of the infection (2–3 months old pigs) to the fundic gland zone in the more chronic phase of the infection (adults sows) in combination with upregulated expressions of IL-8 and IL-1β. Taken together, these findings suggest that shortly after colonization, the immune response is suboptimal, contributing to the persistence of *H. suis* infection. Later a more pronounced immune response is seen, which may result in lower numbers of *H. suis* bacteria in that stomach region. Indeed, although the prevalence of *H. suis* was highest in adult sows, the average number of *H. suis* bacteria per mg tissue decreased with age, as was also observed in other studies [8]. The presence of a specific Treg/Th17 response should be confirmed in future studies, where the expansion of Treg and Th17 cells is directly assessed by the use of staining or flow cytometry.

Interestingly, the expression of CXCL13 was upregulated in *H. suis*-infected pigs of each age group. Since this chemokine attracts B-lymphocytes, its upregulation may be important for the development of a specific local immune response towards *H. suis,* but this requires further research [11]. The upregulation of CXCL13 has also been demonstrated in *H. suis* infected mice [21] and has been linked with the development of mucosa associated lymphoid tissue (MALT)-lymphomas in *Helicobacter* sp. infected human patients [21]. MALT-lymphoma lesions were not detected in the present study and, as far as we know, have not been described in pigs.

In 2–3 months old pigs, the average number of *H. suis* bacteria was the highest in the pyloric gland zone, indicating that *H. suis* colonization starts in this stomach region, as already suggested by others [22]. In adult sows, the average number of *H. suis* bacteria was the highest in the fundic gland zone, indicating a shift in colonization to that region in animals infected during longer periods of time, which is similar to the findings of Hellemans et al. [22]. It appears that when *H. suis* colonizes the stomach epithelium, it triggers an innate immune response in that region, characterized by upregulated expression of the pro-inflammatory cytokines IL-8 and IL-1β in the pyloric gland zone of 2–3 months old pigs and in the fundic gland zone of adult sows.

Severe hyperkeratosis and erosions were only seen in the *H. suis* infected 6–8 months old pigs and not in non-infected pigs of this age group. In adult sows, ulceration was also mainly found in the *H. suis*-positive animals, although this was not significantly different from the *H. suis*-negative group, which may be due to the low number of non-infected sows available. In this field study, interpretation of results is further complicated by variation between herds of other factors that may play a role in development of gastric pathologies such as diet, feeding strategy and management [1]. Nevertheless, our findings provide further evidence that *H. suis* may be one of the factors playing a role in the pathogenesis of gastric ulceration in pigs. A similar conclusion was drawn from results of an experimental infection study in pigs [5]. Interestingly, severe lesions in the *Pars œsophagea* were more frequently found in adult sows compared to the other age groups, indicating that ulceration is a long-term process which may affect the majority of the adult pigs.

It is not yet clear how exactly *H. suis* might influence ulcer development in the *Pars œsophagea*, but alterations in gastric acid secretion might be involved. No clear effects on the markers for gastric acid secretion or number of parietal cells, D-cells and G-cells, and no lesions in the *Pars œsophagea* were detected in the *H. suis*-infected 2–3 months old pigs (acute phase of infection). In a later phase of infection (6–8 months *H. suis* infected pigs), markers for gastric acid secretion were downregulated, genes encoding somatostatin were upregulated and the number of G-cells was decreased, indicating inhibition of gastric acid secretion. In this age group, lesions in the *Pars œsophagea* were present in several animals. The prevalence of severe lesions was extremely high in *H. suis* infected adult sows (chronic phase of infection). Markers for gastric acid secretion were upregulated and the number of G-cells was increased in this age group, indicating increased gastric acid secretion. We hypothesize that decreased gastric acid secretion in the glandular part of the stomach may affect the composition of the *Pars œsophageal* microbiota which may affect development of lesions in this non-glandular part of the stomach. Indeed, higher numbers of a recently described *Fusobacterium* species, designated *F. gastrosuis,* were detected in the *Pars œsophagea* of *H. suis*-infected 6–8 months old pigs than in non-infected pigs of the same age group [23]. Increased production of gastric acid during the chronic phase of infection might further aggravate severity of lesions in this stomach region, which is not protected by mucus. Further studies in which for instance the gastric microbiota and pH are determined in *H. suis*-infected and non-infected pigs, are necessary to confirm or reject this hypothesis.

Several mechanisms might be involved in altered gastric acid secretion in *H. suis* infected animals. A clear parietal cell loss, as described in *H. suis*-infected Mongolian gerbils and mice [9, 24], was not seen in the *H. suis*-infected pigs, although small differences in the number of these cells between the infected and non-infected animals cannot be excluded since counting was impossible in the fundic gland zone. The expression of genes encoding H+/K+ ATPase was, however, altered. As this enzyme is typically associated with gastric acid production by parietal cells, this shows that the function of these cells was affected. This is also indicated by altered expression of H2-, M3- and CCK-B receptors, although these receptors are also found on enterochromaffin cells, which were not studied here. The exact mechanism behind the effect of a *H. suis* infection on gastric acid secretion by parietal cells is not clear and requires further studies. Since *H. suis* is often found in close proximity to these host cells, a direct effect of this bacterium on the parietal cells might be involved. Indirect effects probably also play a role since the number of G-cells and/or the expression of gastrin was decreased or enhanced in pigs with downregulated and upregulated expression of H+/K+ ATPase, respectively. In *H. pylori* infections, increased gastric acid secretion has been associated with increased expression of genes encoding IL-8 and IL-1β [25–29]. Expression of genes encoding these cytokines was upregulated in the fundic gland zone of adult sows with upregulated expression of genes encoding H+/K+ ATPase. Literature dealing with the effect of IL-1β is, however, controversial as Beales and Calam [30] demonstrated that IL-1β inhibits acid secretion in cultured parietal cells.

In *H. suis* infected 6–8 months old pigs and adult sows, expression of genes encoding Sonic Hedgehog was upregulated in the fundic and pyloric gland zone. Since Sonic Hedgehog is involved in the regeneration of damaged epithelium [31], this may indicate a compensation for epithelial loss induced by *H. suis* in these gastric regions. Disruption of the gastric epithelium, followed by regeneration was further suggested by the downregulated expression of genes encoding claudin 18, an important tight junction protein of the stomach [32], in *H. suis*-infected 6–8 months old pigs and its upregulation in adult sows. The increased IL-10 and IL-17A transcript levels in *H. suis*-infected adult sows may have promoted the regeneration of the gastric epithelium as well, as both cytokines are associated with intestinal barrier restoration [33].

**Figure 5:**
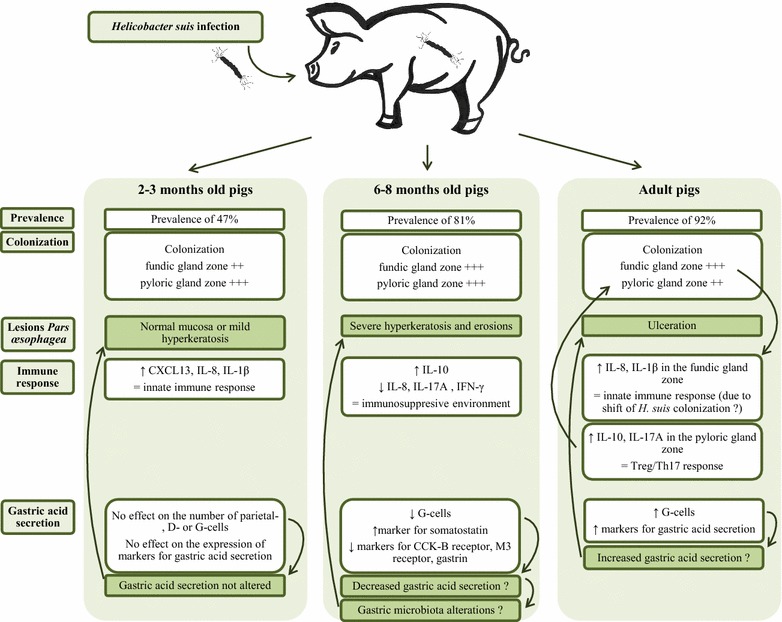
**A diagram summarizing the main conclusions from the present study.** An overview of the effects of a *H. suis* infection on gastric acid secretion and lesion development in pigs of different ages. *H. suis* affects the expression of markers for gastric acid secretion and inflammation and these effects may differ, depending on the infection phase.

To summarize, we revealed an increased prevalence of *H. suis* and a shift of colonization towards the fundic gland zone in adult sows, while the number of *H. suis* bacteria per mg tissue decreased with age. Gastric erosion and ulceration were more frequently detected in *H. suis*-infected pigs. During the more acute phase of the infection, an innate immune response was indicated to be present, followed by a Treg and Th17 response in pigs colonized during longer periods of time. While no clear alterations in the markers for gastric acid secretion were detected in 2–3 months old pigs, a decrease and increase were found in 6–8 months old pigs and adult sows, respectively. These results indicate that *H. suis* affects the expression of markers for gastric acid secretion and inflammation and indicate that these effects differ, depending on the infection phase. An overview of the main results and conclusions of the present study is presented in Figure 5.

## Additional files

**Additional file 1.** **List of primers used in quantitative RT-PCR for gene expression analysis of markers for gastric acid secretion and inflammation.**

**Additional file 2.** **The number of** ***H. suis*** **bacteria in the different stomach regions of 2–3 months old pigs (A), 6–8 months old pigs (B) and adult sows (C).** Data are shown as log10 values of the average number of *H. suis* bacteria per mg tissue with standard deviation. Statistical differences were calculated using the non-parametric Kruskal–Wallis H test. *, *p* < 0.01; **, *p* < 0.001 significant differences between the stomach regions.

**Additional file 3.** **General overview of the average scores of infiltration with inflammatory cells and lymphoid follicle formation in the fundic and pyloric gland zone of pigs of different ages.** Gastritis was scored based on infiltration with inflammatory cells/lymphoid follicle formation, with score 0 = absence of infiltration/absence of lymphoid aggregates, 1 = mild infiltration/small number of lymphoid aggregates (*n* < 5), 2 = moderate infiltration/large number of lymphoid aggregates (*n* > 5) or presence of 1 organized lymphoid follicle, 3 = marked infiltration/at least 2 organized lymphoid follicles, *n* = total number of investigated pigs’ stomachs per age group. The data are shown as the average of the administered scores with standard deviation.

**Additional file 4.** **Microscopic visualization of the parietal cells (A), D-cells (B) and G-cells (C–D) in the porcine stomach using immunohistochemistry.** (A) H+/K+ ATPase staining of the fundic gland zone of a *H. suis*-positive adult sow, showing parietal cells (brown). No clear parietal cell loss was detected. Original magnification ×100. (B) Somatostatin staining of the pyloric gland zone of a *H. suis*-positive adult sow, showing D-cells (brown). Original magnification ×200. (C) Gastrin staining of the pyloric gland zone of a *H. suis*-positive adult sow, showing G-cells (brown). Original magnification ×200. (D) Gastrin staining of the pyloric gland zone of a *H. suis*-negative sow, showing G-cells (brown). Original magnification ×200. The number of G-cells in the *H. suis*-negative sow (D) is lower than observed in the *H. suis*-positive sow (C).

**Additional file 5.** **Overview of the relative fold changes of altered markers for inflammation in** ***H. suis*** **infected pigs of different ages.** The data are presented as fold changes in gene expression normalized to 3 reference genes and relative to the *H. suis*-negative control group which is considered as 1. The fold changes are shown as means with the standard error of the mean. Statistical differences were calculated using the non-parametric Kruskal–Wallis H test. A *p* value lower than 0.05 is considered to be significant.

**Additional file 6.** **Overview of important correlations between markers for inflammation and the number of** ***H. suis*** **bacteria in pigs of different ages.** *r* = Pearson correlation coefficient, calculated using SPSS Statistics 24^®^. A *r*-value close to 1 indicates a strong, positive correlation, whereas a *r*-value of −1 indicates a strong, negative correlation. A *p*-value lower than 0.05 is considered to be significant.

**Additional file 7.** **Overview of relative fold changes of altered markers for gastric acid secretion in** ***H. suis*** **-infected pigs of different ages.** The data are presented as fold changes in gene expression normalized to 3 reference genes and relative to the*H. suis*-negative control group which is considered as 1. The fold changes are shown as means with the standard error of the mean. Statistical differences were calculated using the non-parametric Kruskal–Wallis H test. A *p*-value lower than 0.05 is considered to be significant.

**Additional file 8.** **Overview of important correlations between markers for gastric acid secretion and the number of** ***H. suis*** **bacteria in pigs of different ages.** *r* = Pearson correlation coefficient, calculated using SPSS Statistics 24^®^. A *r*-value close to 1 indicates a strong, positive correlation, whereas a *r*-value of -1 indicates a strong, negative correlation. *P*-values lower than 0.05 are considered to be significant.

**Additional file 9.** **Correlations of altered markers for gastric acid secretion with the number of** ***H. suis*** **bacteria and with the altered markers for inflammation in** ***H. suis*** **-infected pigs of different age groups.** *r* = Pearson correlation coefficient, calculated using SPSS Statistics 24^®^. A *r*-value close to 1 indicates a strong, positive correlation, whereas a *r*-value of −1 indicates a strong, negative correlation. *P*-values lower than 0.05 are considered to be significant./= no clear correlation, yes = correlation with *H. suis* colonization rate (see Additional files 6, 8 for the *r* and *p*-values).
