# Supplementary material for: Helicobacter suis induces changes in gastric inflammation and acid secretion markers in pigs of different ages
Source: Vet Res. 2017 Jun 15;48:34. doi: 10.1186/s13567-017-0441-6 (PMC5473008; doi:10.1186/s13567-017-0441-6)
Supplement: Supplementary file 1 — Additional file 1. List of primers used in quantitative RT-PCR for gene expression analysis of markers for gastric acid secretion and inflammation. [file 13567_2017_441_MOESM1_ESM.docx]

**Additional file 1**: List of primers used in quantitative RT-PCR for gene expression analysis of markers for gastric acid secretion and inflammation.

| **Primer** | **Sequence** | **Reference** |
| --- | --- | --- |
| HPRT-forward | 5’-GTG ATA GAT CCA TTC CTA TGA CTG TAG A-3’ | [35] |
| HPRT-reverse | 5’-TGA GAG ATC ATC TCC ACC AAT TAC TT-3’ | [35] |
| Cyc5-forward | 5’-CCT GAA CAT ACG GGT CCT G-3’ | [36] |
| Cyc5-reverse | 5’-AAC TGG GAA CCG TTT GTG TTG-3’ | [36] |
| ACTB-forward | 5’-CTC TTC CAG CCC TCC TTC CT-3’ | [37] |
| ACTB-reverse | 5’-GCG TAG AGG TCC TTC TTC CTG ATG T-3’ | [37] |
| H+/K+ ATPase-forward | 5’-GCA TAT GAG AAG GCC GAG AG-3’ | [38] |
| H+/K+ ATPase-reverse | 5’-TGG CCG TGA AGT AGT CAG TG-3’ | [38] |
| Sonic Hedgehog-forward | 5’-TGA CCC CTT TAG CCT ACA AGC A-3’ | [16] |
| Sonic Hedgehog-reverse | 5’-TGG GGG TGA GTT CCT TAA ATC G-3’ | [16] |
| Claudin 18-forward | 5’-CAT GGG TGG GAT GGT GCA GA-3’ | [16] |
| Claudin 18-reverse | 5’-CGG CAA GCG ATG CAC ATC AT-3’ | [16] |
| KCNQ1-forward | 5’-CTC CGT GGT CTT CAT CCA C-3’ | [39] |
| KCNQ1-reverse | 5’-GTA GCT GCC GAA CTC CAC TT-3’ | [39] |
| Gastrin-forward | 5’-TCC TCA GCA CTG CGG CGG-3’ | [40] |
| Gastrin-reverse | 5’-ATG GAG GAG GAA GAA GAA GC-3’ | [40] |
| Cholinergic muscarinic M3 receptor-forward | 5’-AAC AAT GAT GCT GCT GCC-3’ | [41] |
| Cholinergic muscarinic M3 receptor-reverse | 5’-GTG ATC TGA CTT CTG GTC TTC-3’ | [41] |
| Somatostatin-forward | 5’-GTC CTG GCT **C**TG GGC GGT GTC A-3’ | Adjusted from [42] |
| Somatostatin-reverse | 5’-TGC AGC TCC AGC CTC AT**T** TC**A** T-3’ | Adjusted from [42] |
| Histamine H2 receptor-forward | 5’-CCA CCA TCA GGG AGC ACA A-3’ | [43] |
| Histamine H2 receptor-reverse | 5’-AGG G**G**A ACC AGC AGA TGA TGA A-3’ | Adjusted from [43] |
| CCK-B receptor-forward | 5’-CGC CAT CTG CCG ACC ACT GC-3’ | [16] |
| CCK-B receptor-reverse | 5’-TTG GCT GTC GCT GTC ACT GT-3’ | [16] |
| IL-1β-forward | 5’-GGC CGC CAA GAT ATA ACT GA-3’ | [44] |
| IL-1β-reverse | 5’-GGA CCT CTG GGT ATG GCT TTC-3’ | [44] |
| IL-4-forward | 5’-GAC ACA AGT GCG ACA TCA CC-3’ | [45] |
| IL-4-reverse | 5’-AGC TCC ATG CAC GAG TTC TT-3’ | [45] |
| IL-8-forward | 5’-TTC GAT GCC AGT GCA TAA ATA-3’ | [44] |
| IL-8-reverse | 5’-CTG TAC AAC CTT CTG CAC CCA-3’ | [44] |
| IL-10-forward | 5’-GAT ATC AAG GAG CAC GTG AAC TC-3’ | [37] |
| IL-10-reverse | 5’-GAG CTT GCT AAA GGC ACT CTT C-3’ | [37] |
| IL-17A-forward | 5’-CTC TCG TGA AGG CGG GAA TC-3’ | [46] |
| IL-17A-reverse | 5’-GTA ATC TGA GGG CCG TCT GG-3’ | [46] |
| IFN-γ-forward | 5’-AGG TTC CTA AAT GGT AGC TCT GGG-3’ | [37] |
| IFN-γ-reverse | 5’-AGT TCA CTG ATG GCT TTG CGC T-3’ | [37] |
| CXCL13-forward | 5’-GAT CTT TCC CAT CCA AGC AA-3’ | [45] |
| CXCL13-reverse | 5’-AAC GCA AAT GGT CAG TAG GG-3’ | [45] |
