# Supplementary material for: Helicobacter suis induces changes in gastric inflammation and acid secretion markers in pigs of different ages
Source: Vet Res. 2017 Jun 15;48:34. doi: 10.1186/s13567-017-0441-6 (PMC5473008; doi:10.1186/s13567-017-0441-6)
Supplement: Supplementary file 2 — Additional file 2. The number of H. suis bacteria in the different stomach regions of 2–3 months old pigs (A), 6–8 months old pigs (B) and adult sows (C). Data are shown as log10 values of the average number of H. suis bacteria per mg tissue with standard deviation. Statistical differences were calculated using the non-parametric Kruskal–Wallis H test. *, p < 0.01; **, p < 0.001 significant differences between the stomach regions. [file 13567_2017_441_MOESM2_ESM.docx]

|  | **  **  **  ** |
| --- | --- |
|  |  |
