# Supplementary material for: Helicobacter suis induces changes in gastric inflammation and acid secretion markers in pigs of different ages
Source: Vet Res. 2017 Jun 15;48:34. doi: 10.1186/s13567-017-0441-6 (PMC5473008; doi:10.1186/s13567-017-0441-6)
Supplement: Supplementary file 3 — Additional file 3. General overview of the average scores of infiltration with inflammatory cells and lymphoid follicle formation in the fundic and pyloric gland zone of pigs of different ages. Gastritis was scored based on infiltration with inflammatory cells/lymphoid follicle formation, with score 0 = absence of infiltration/absence of lymphoid aggregates, 1 = mild infiltration/small number of lymphoid aggregates (n < 5), 2 = moderate infiltration/large number of lymphoid aggregates (n > 5) or presence of 1 organized lymphoid follicle, 3 = marked infiltration/at least 2 organized lymphoid follicles, n = total number of investigated pigs’ stomachs per age group. The data are shown as the average of the administered scores with standard deviation. [file 13567_2017_441_MOESM3_ESM.docx]

**Additional file 3**: General overview of the average scores of lymphoid infiltration and lymphoid follicle formation in the fundic and pyloric gland zone of pigs of different ages.

| **Age group** | **Lymphoid infiltration** | | **Lymphoid follicles** | |
| --- | --- | --- | --- | --- |
|  | **Fundic gland zone** | **Pyloric gland zone** | **Fundic gland zone** | **Pyloric gland zone** |
| **2-3 months old (n=34)** | 1.6 ± 0.3 | 2.0 ± 0.7 | 1.0 ± 0.5 | 1.5 ± 0.4 |
| *H. suis* positive (n=16) | 1.6 ± 0.3 | 2.0 ± 0.6 | 1.3 ± 0.4 | 1.7 ± 0.3 |
| *H. suis* negative (n=18) | 1.5 ± 0.5 | 2.1 ± 0.4 | 1.0 ± 0.5 | 1.3 ± 0.4 |
| **6-8 months old (n=68)** | 1.8 ± 0.6 | 2.1 ± 0.7 | 1.0 ± 0.6 | 1.7 ± 1.0 |
| *H. suis* positive (n=55) | 1.7 ± 0.6 | 2.1 ± 0.6 | 1.0 ± 0.6 | 1.8 ± 1.0 |
| *H. suis* negative (n=13) | 2.1 ± 0.6 | 1.9 ± 0.8 | 1.2 ± 0.4 | 1.3 ± 0.9 |
| **Adult sows (n=60)** | 1.7 ± 0.6 | 2.1 ± 0.7 | 1.2 ± 0.7 | 1.6 ± 0.6 |
| *H. suis* positive (n=55) | 1.7 ± 0.7 | 2.1 ± 0.7 | 1.3 ± 0.7 | 1.6 ± 0.6 |
| *H. suis* negative (n=5) | 1.8 ± 0.4 | 2.4 ± 0.5 | 1.0 ± 0.7 | 1.2 ± 0.4 |

Gastritis was scored based on infiltration with inflammatory cells / lymphoid follicle formation, with score 0 = absence of infiltration / absence of lymphoid aggregates, 1 = mild infiltration / small number of lymphoid aggregates (n < 5), 2 = moderate infiltration / large number of lymphoid aggregates (n > 5) or presence of 1 organized lymphoid follicle, 3 = marked infiltration / at least 2 organized lymphoid follicles, n = total number of investigated pigs’ stomachs per age group. The data are shown as the average of the administered scores with standard deviation.
