# Supplementary figures and images for: Helicobacter suis induces changes in gastric inflammation and acid secretion markers in pigs of different ages
Source: Vet Res. 2017 Jun 15;48:34. doi: 10.1186/s13567-017-0441-6 (PMC5473008; doi:10.1186/s13567-017-0441-6)

| 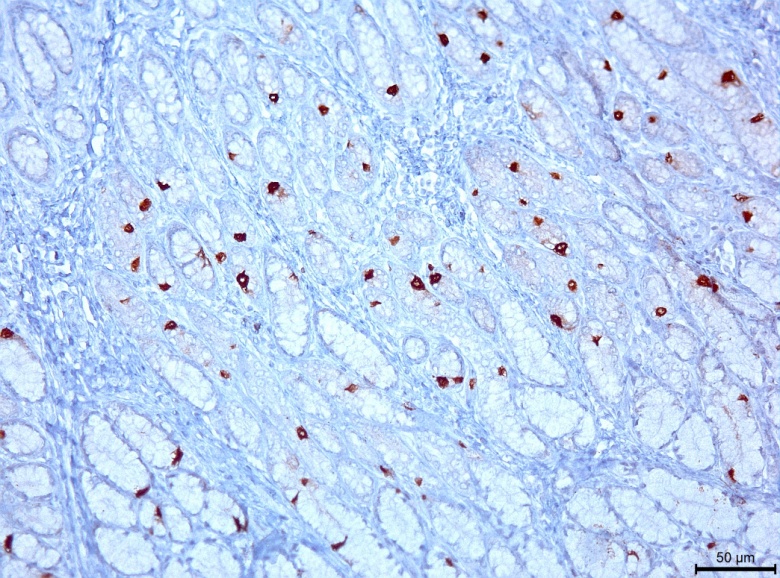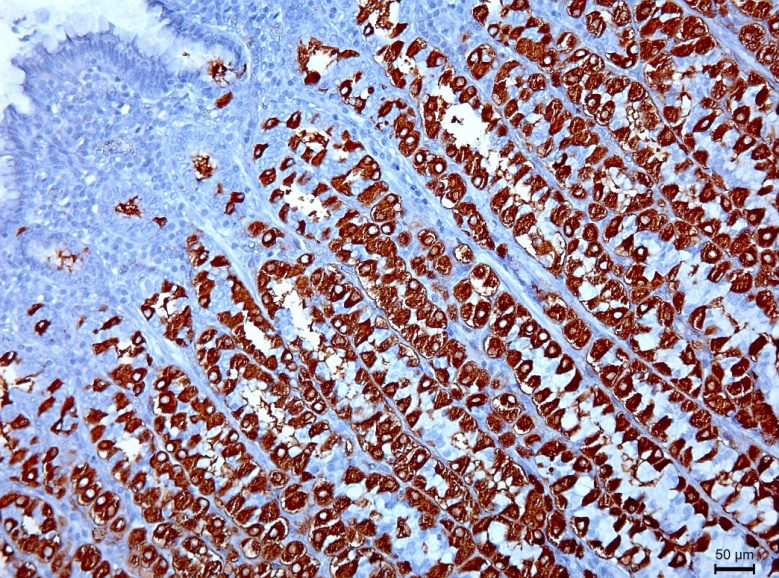  **C**  **A** | 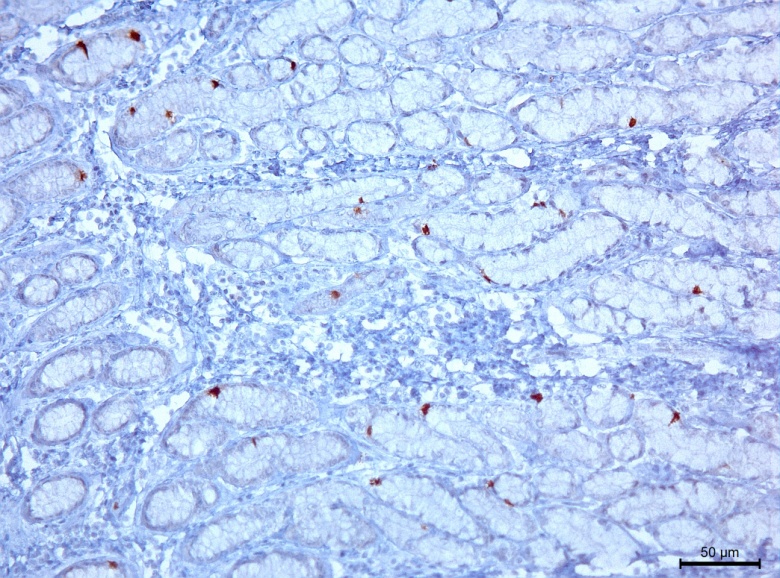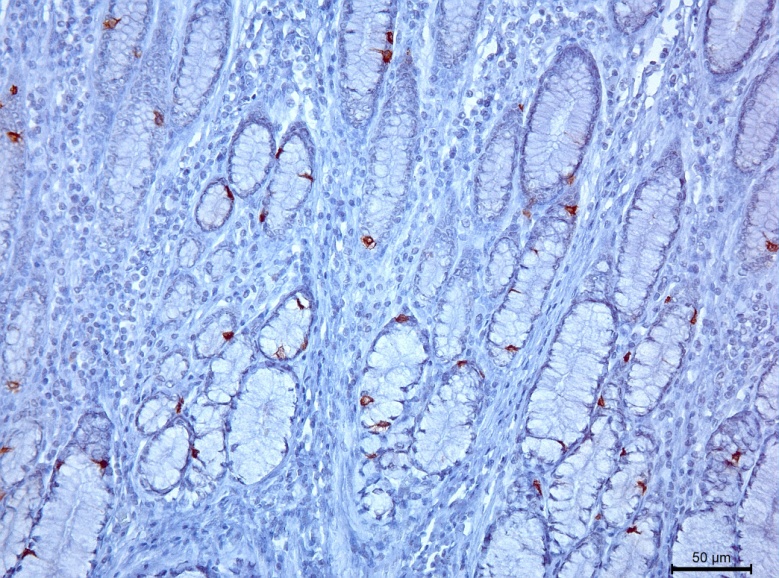  **D**  **B** |
| --- | --- |

Supplement: Supplementary file 4 — Additional file 4. Microscopic visualization of the parietal cells (A), D-cells (B) and G-cells (C–D) in the porcine stomach using immunohistochemistry. (A) H+/K+ ATPase staining of the fundic gland zone of a H. suis-positive adult sow, showing parietal cells (brown). No clear parietal cell loss was detected. Original magnification ×100. (B) Somatostatin staining of the pyloric gland zone of a H. suis-positive adult sow, showing D-cells (brown). Original magnification ×200. (C) Gastrin staining of the pyloric gland zone of a H. suis-positive adult sow, showing G-cells (brown). Original magnification ×200. (D) Gastrin staining of the pyloric gland zone of a H. suis-negative sow, showing G-cells (brown). Original magnification ×200. The number of G-cells in the H. suis-negative sow (D) is lower than observed in the H. suis-positive sow (C). [file 13567_2017_441_MOESM4_ESM.docx]
