# Supplementary material for: Helicobacter suis induces changes in gastric inflammation and acid secretion markers in pigs of different ages
Source: Vet Res. 2017 Jun 15;48:34. doi: 10.1186/s13567-017-0441-6 (PMC5473008; doi:10.1186/s13567-017-0441-6)
Supplement: Supplementary file 5 — Additional file 5. Overview of the relative fold changes of altered markers for inflammation in H. suis infected pigs of different ages. The data are presented as fold changes in gene expression normalized to 3 reference genes and relative to the H. suis-negative control group which is considered as 1. The fold changes are shown as means with the standard error of the mean. Statistical differences were calculated using the non-parametric Kruskal–Wallis H test. A p value lower than 0.05 is considered to be significant. [file 13567_2017_441_MOESM5_ESM.docx]

**Additional file 5:** Overview of the relative fold changes of altered markers for inflammation in *H. suis*-infected pigs of different ages.

| **Age group** | **Gene** | **Relative fold change** | **P-value** |
| --- | --- | --- | --- |
| **2-3 months old pigs** |  |  |  |
| Fundic gland zone | CXCL13 | 2.59 ± 0.52 | 0.027 |
| Pyloric gland zone | IL-8 | 2.37 ± 0.27 | 0.001 |
|  | IL-17A | 0.81 ± 0.15 | 0.039 |
|  | IL-1β | 2.03 ± 0.28 | 0.034 |
|  | CXCL13 | 19.21 ± 3.78 | < 0.001 |
| **6-8 months old pigs** |  |  |  |
| Fundic gland zone | IL-4 | 3.15 ± 0.68 | 0.183 |
|  | IL-8 | 0.49 ± 0.16 | 0.040 |
|  | IL-10 | 3.77 ± 0.90 | 0.047 |
|  | IL-17A | 0.75 ± 0.25 | 0.029 |
|  | IFN-γ | 0.11 ± 0.02 | < 0.001 |
|  | CXCL13 | 6.49 ± 2.20 | 0.011 |
| Pyloric gland zone | IL-4 | 2.08 ± 0.42 | 0.095 |
|  | IL-17A | 2.63 ± 0.58 | 0.128 |
|  | CXCL13 | 3.99 ± 0.17 | 0.196 |
|  | IFN-γ | 0.56 ± 0.17 | 0.005 |
| **Adult sows** |  |  |  |
| Fundic gland zone | IL-8 | 5.23 ± 1.27 | 0.018 |
|  | IL-10 | 1.75 ± 0.32 | 0.243 |
|  | IL-17 | 2.02 ± 0.26 | 0.148 |
|  | IL-1β | 6.74 ± 1.48 | 0.037 |
| Pyloric gland zone | IL-10 | 1.87 ± 0.22 | 0.019 |
|  | IL-17A | 2.00 ± 0.21 | 0.042 |
|  | IFN-γ | 3.50 ± 0.78 | 0.371 |
|  | CXCL13 | 2.16 ± 0.53 | 0.169 |

The data are presented as fold changes in gene expression normalized to 3 reference genes and relative to the *H. suis*-negative control group which is considered as 1. The fold changes are shown as means with the standard error of the mean. Statistical differences were calculated using the non-parametric Kruskal-Wallis H test SPSS statistics 24®. A P-value lower than 0.05 is considered to be significant.
