# Supplementary material for: Helicobacter suis induces changes in gastric inflammation and acid secretion markers in pigs of different ages
Source: Vet Res. 2017 Jun 15;48:34. doi: 10.1186/s13567-017-0441-6 (PMC5473008; doi:10.1186/s13567-017-0441-6)
Supplement: Supplementary file 6 — Additional file 6. Overview of important correlations between markers for inflammation and the number of H. suis bacteria in pigs of different ages. r = Pearson correlation coefficient, calculated using SPSS Statistics 24®. A r-value close to 1 indicates a strong, positive correlation, whereas a r-value of −1 indicates a strong, negative correlation. A p-value lower than 0.05 is considered to be significant. [file 13567_2017_441_MOESM6_ESM.docx]

**Additional file 6:** Overview of important correlations between markers for inflammation and the number of *H. suis* bacteria in pigs of different ages.

|  | **Fundic gene expression and the number of *H. suis* bacteria in the fundic gland zone** | | **Antral gene expression and the number of *H. suis* bacteria in the pyloric gland zone** | |
| --- | --- | --- | --- | --- |
| **Age group** | **Gene** |  | **Gene** |  |
| **2-3 months old pigs** | CXCL13 | r = 0.307  P = 0.023 | IL-8 | r = 0.514  P < 0.001 |
|  |  |  | IL-17A | r = -0.335  P = 0.011 |
|  |  |  | IL-1β | r = 0.371  P = 0.005 |
|  |  |  | CXCL13 | r = 0.584  P < 0.001 |
| **6-8 months old pigs** | IL-4 | r = 0.224  P = 0.120 | IL-4 | r = 0.259  P = 0.08 |
|  | IL-8 | r = -0.279  P = 0.059 | IL-17A | r = 0.197  P = 0.176 |
|  | IL-10 | r = 0.288  P = 0.046 | IFN-γ | r = -0.339  P = 0.017 |
|  | IL-17A | r = -0.315  P = 0.027 | CXCL13 | r = 0.293  P = 0.050 |
|  | IFN-γ | r = -0.465  P = 0.002 |  |  |
|  | CXCL13 | r = 0.419  P = 0.004 |  |  |
| **Adult sows** | IL-8 | r = 0.371  P = 0.013 | IL-8 | r = 0.320  P = 0.039 |
|  | IL-10 | r = 0.192  P = 0.187 | IL-10 | r = 0.255  P = 0.091 |
|  | IL-17A | r = 0.226  P = 0.120 | IL-17A | r = 0.277  P = 0.066 |
|  | IL-1β | r = 0.185  P = 0.213 | IFN-γ | r = 0.380  P = 0.012 |
|  |  |  | CXCL13 | r = 0.255  P = 0.091 |

r = Pearson correlation coefficient, calculated using SPSS Statistics 24®. A r-value close to 1 indicates a strong, positive correlation, whereas a r-value of -1 indicates a strong, negative correlation. P-values lower than 0.05 are considered to be significant.
