# Supplementary material for: Helicobacter suis induces changes in gastric inflammation and acid secretion markers in pigs of different ages
Source: Vet Res. 2017 Jun 15;48:34. doi: 10.1186/s13567-017-0441-6 (PMC5473008; doi:10.1186/s13567-017-0441-6)
Supplement: Supplementary file 7 — Additional file 7. Overview of relative fold changes of altered markers for gastric acid secretion in H. suis -infected pigs of different ages. The data are presented as fold changes in gene expression normalized to 3 reference genes and relative to theH. suis-negative control group which is considered as 1. The fold changes are shown as means with the standard error of the mean. Statistical differences were calculated using the non-parametric Kruskal–Wallis H test. A p-value lower than 0.05 is considered to be significant. [file 13567_2017_441_MOESM7_ESM.docx]

**Additional file 7:** Overview of relative fold changes of altered markers for gastric acid secretion in *H. suis-*infected pigs of different ages.

| **Age group** | **Gene** | **Relative fold change** | **P-value** |
| --- | --- | --- | --- |
| **2-3 months old pigs** |  |  |  |
| Fundic gland zone | KCNQ1 | 1.98 ± 0.33 | 0.088 |
| Pyloric gland zone | M3-receptor | 0.77 ± 0.09 | 0.027 |
|  | Somatostatin | 0.83 ± 0.11 | 0.091 |
| **6-8 months old pigs** |  |  |  |
| Fundic gland zone | Claudin 18 | 0.61 ± 0.11 | 0.022 |
|  | Gastrin | 0.70 ± 0.16 | 0.040 |
|  | M3 receptor | 0.62 ± 0.08 | 0.002 |
|  | CCK-B receptor | 0.51 ± 0.32 | 0.004 |
| Pyloric gland zone | H+/K+ ATPase | 3.44 ± 0.83 | 0.106 |
|  | Sonic Hedgehog | 8.95 ± 2.17 | 0.048 |
|  | Somatostatin | 12.13 ± 2.85 | 0.007 |
| **Adult sows** |  |  |  |
| Fundic gland zone | H+/K+ ATPase | 4.17 ± 0.67 | 0.049 |
|  | Sonic Hedgehog | 2.03 ± 0.39 | 0.477 |
|  | Claudin 18 | 3.20 ± 0.29 | 0.002 |
|  | KCNQ1 | 3.02 ± 0.43 | 0.012 |
|  | Gastrin | 5.81 ± 0.63 | < 0.001 |
|  | Somatostatin | 2.12 ± 0.39 | 0.447 |
|  | H2 receptor | 5.27 ± 1.10 | 0.019 |
|  | CCK-B receptor | 1.89 ± 0.18 | 0.049 |
| Pyloric gland zone | H+/K+ ATPase | 10.44 ± 2.83 | 0.002 |
|  | Sonic Hedgehog | 2.46 ± 0.27 | 0.060 |
|  | Claudin 18 | 2.13 ± 0.19 | 0.012 |
|  | Gastrin | 1.75 ± 0.21 | 0.068 |
|  | M3 receptor | 0.66 ± 0.12 | 0.049 |
|  | Somatostatin | 2.02 ± 0.20 | 0.071 |
|  | H2 receptor | 12.48 ± 3.07 | 0.015 |
|  | CCK-B receptor | 1.96 ± 0.17 | 0.012 |

The data are presented as fold changes in gene expression normalized to 3 reference genes and relative to the *H. suis*-negative control group which is considered as 1. The fold changes are shown as means with the standard error of the mean. Statistical differences were calculated using the non-parametric Kruskal-Wallis H test SPSS statistics 24®. A P-value lower than 0.05 is considered to be significant.
