# Supplementary material for: Helicobacter suis induces changes in gastric inflammation and acid secretion markers in pigs of different ages
Source: Vet Res. 2017 Jun 15;48:34. doi: 10.1186/s13567-017-0441-6 (PMC5473008; doi:10.1186/s13567-017-0441-6)
Supplement: Supplementary file 8 — Additional file 8. Overview of important correlations between markers for gastric acid secretion and the number of H. suis bacteria in pigs of different ages. r = Pearson correlation coefficient, calculated using SPSS Statistics 24®. A r-value close to 1 indicates a strong, positive correlation, whereas a r-value of -1 indicates a strong, negative correlation. P-values lower than 0.05 are considered to be significant. [file 13567_2017_441_MOESM8_ESM.docx]

**Additional file 8:** Overview of important correlations between markers for gastric acid secretion and the number of *H. suis* bacteria in pigs of different ages.

|  | **Gene expression and the number of *H. suis* bacteria in the fundic gland zone** | | **Gene expression and the number of *H. suis* bacteria in the pyloric gland zone** | |
| --- | --- | --- | --- | --- |
| **Age group** | **Gene – stomach region** |  | **Gene – stomach region** |  |
| **2-3 months old pigs** | KNCQ1 - fundic gland zone | r = 0.401  P = 0.002 | Somatostatin - pyloric gland zone | r = -0.287  P = 0.029 |
|  | Somatostatin - pyloric gland zone | r = -0.313  P = 0.018 | M3 receptor - pyloric gland zone | r = -0.173  P = 0.188 |
| **6-8 months old pigs** | Claudin 18 – fundic gland zone | r = -0.327  P = 0.022 | Claudin 18 – fundic gland zone | r = -0.285  P = 0.045 |
|  | Gastrin – fundic gland zone | r = -0.279  P = 0.066 | Gastrin – fundic gland zone | r = -0.272  P = 0.074 |
|  | M3 receptor – fundic gland zone | r = -0.445  P = 0.002 | M3 receptor – fundic gland zone | r = -0.356  P = 0.012 |
|  | CCK-B - fundic gland zone | r = -0.469  P = 0.001 | CCK-B - fundic gland zone | r = -0.392  P = 0.006 |
|  | H+/K+ ATPase – pyloric gland zone | r = 0.293  P = 0.045 | H+/K+ ATPase – pyloric gland zone | r = 0.245  P = 0.098 |
|  | Gastrin – pyloric gland zone | r = 0.250  P = 0.089 | Gastrin – pyloric gland zone | r = 0.338  P = 0.022 |
|  | Sonic Hedgehog – pyloric gland zone | r = 0.366  P = 0.015 | Sonic Hedgehog – pyloric gland zone | r = 0.402  P = 0.007 |
|  | Somatostatin – pyloric gland zone | r = 0.323  P = 0.026 | Somatostatin – pyloric gland zone | r = 0.418  P = 0.004 |
| **Adult sows** | Claudin 18 – fundic gland zone | r = 0.260  P = 0.074 | H+/K+ ATPase – fundic gland zone | r = 0.410  P = 0.007 |
|  | CCK-B receptor – pyloric gland zone | r = 0.263  P = 0.070 | KCNQ1 – fundic gland zone | r = 0.365  P = 0.015 |
|  |  |  | Sonic Hedgehog – fundic gland zone | r = 0.368  P = 0.016 |
|  |  |  | CCK-B receptor – fundic gland zone | r = 0.259  P = 0.086 |
|  |  |  | CCK-B receptor - pyloric gland zone | r = 0.269  P = 0.074 |

r = Pearson correlation coefficient, calculated using SPSS Statistics 24®. A r-value close to 1 indicates a strong, positive correlation, whereas a r-value of -1 indicates a strong, negative correlation. P-values lower than 0.05 are considered to be significant.
