# Supplementary material for: Helicobacter suis induces changes in gastric inflammation and acid secretion markers in pigs of different ages
Source: Vet Res. 2017 Jun 15;48:34. doi: 10.1186/s13567-017-0441-6 (PMC5473008; doi:10.1186/s13567-017-0441-6)
Supplement: Supplementary file 9 — Additional file 9. Correlations of altered markers for gastric acid secretion with the number of H. suis bacteria and with the altered markers for inflammation in H. suis -infected pigs of different age groups. r = Pearson correlation coefficient, calculated using SPSS Statistics 24®. A r-value close to 1 indicates a strong, positive correlation, whereas a r-value of −1 indicates a strong, negative correlation. P-values lower than 0.05 are considered to be significant./= no clear correlation, yes = correlation with H. suis colonization rate (see Additional files 6, 8 for the r and p-values). [file 13567_2017_441_MOESM9_ESM.docx]

**Additional file 9:** Correlations of altered markers for gastric acid secretion with the number of *H. suis* bacteria and with the altered markers for inflammation in *H. suis-*infected pigs of different age groups.

| **Age group** | **Markers for gastric acid secretion** | **Correlation with the number of *H. suis* bacteria** | | **Correlation with markers for inflammation** | |
| --- | --- | --- | --- | --- | --- |
|  |  | **Fundic gland zone** | **Pyloric gland zone** | **Fundic gland zone** | **Pyloric gland zone** |
| **2-3 months old**  Fundic gland zone | KCNQ1 | r = 0.401; P = 0.002 | / | / | / |
| Pyloric gland zone | M3 receptor | / | r = -0.173; P = 0.188 | / | / |
|  | Somatostatin | r = -0.313; P = 0.018 | r = -0.287; P = 0.029 | CXCL13  r = -0.529; P = 0.042 | IL-1β  r = -0.443; P = 0.086  CXCL13  r = -0.617; P = 0.011 |
| **6-8 months old** |  |  |  |  |  |
| Fundic gland zone | Claudin 18 | r = -0.327; P = 0.022 | r = -0.285; P = 0.045 | IL-8  r = 0.500; P = 0.058  IFN-γ  r = 0.579; P = 0.024 | / |
|  | Gastrin | r = -0.279; P = 0.066 | r = -0.272; P = 0.074 | / | / |
|  | M3 receptor | r = -0.445; P = 0.002 | r = -0.356; P = 0.012 | / | / |
|  | CCK-B receptor | r = -0.469; P = 0.001 | r = -0.392; P = 0.006 | IL-8  r = 0.526; P = 0.044  IL-17A  r = 0.754; P = 0.001  IFN-γ  r = 0.523; P = 0.046 | / |
| Pyloric gland zone | H+/K+ ATPase | r = 0.293; P = 0.045 | r = 0.245; P = 0.098 | / | / |
|  | Sonic Hedgehog | r = 0.366; P = 0.015 | r = 0.402; P = 0.007 | / | / |
|  | Gastrin | r = 0.250; P = 0.089 | r = 0.338; P = 0.022 | / | / |
|  | Somatostatin | r = 0.323; P = 0.026 | r = 0.418; P = 0.004 | IL-17A  r = -0.429; P = 0.098 | / |
| **Adult sows** |  |  |  |  |  |
| Fundic gland zone | H+/K+ ATPase | / | r = 0.410; P = 0.007 | / | / |
|  | Sonic Hedgehog | / | r = 0.368; P = 0.016 | IL-17A  r = 0.575; P = 0.008 | IFN-γ  r = 0.512; P = 0.021 |
|  | Claudin 18 | r = 0.260; P = 0.074 | / |  | IL-17A  r = 0.470; P = 0.037  IFN-γ  r = 0.468; P = 0.037 |
|  | KCNQ1 | / | r = 0.365; P = 0.015 | / | / |
|  | Gastrin | / | / | / | / |
|  | Somatostatin | / | / | / | / |
|  | H2 receptor | / | / | / | / |
|  | CCK-B receptor | / | r = 0.259; P = 0.086 | / | / |
| Pyloric gland zone | H+/K+ ATPase | / | / | IL-10  r = 0.554; P = 0.014 | / |
|  | Sonic Hedgehog | / | / | / | IL-10  r = 0.678; P = 0.001 |
|  | Claudin 18 | / | / | / | IL-10  r = 0.502; P = 0.024 |
|  | Gastrin | / | / | / | IL-10  r = 0.426; P = 0.061 |
|  | M3 receptor | / | / | / | / |
|  | Somatostatin | / | / | / | / |
|  | H2 receptor | / | / | / | / |
|  | CCK-B receptor | r = 0.263; P = 0.070 | r = 0.269; P = 0.074 | / | IL-10  r = 0.454; P = 0.045 |

r = Pearson correlation coefficient, calculated using SPSS Statistics 24®. A r-value close to 1 indicates a strong, positive correlation, whereas a r-value of -1 indicates a strong, negative correlation. P-values lower than 0.05 are considered to be significant. / = no clear correlation, yes = correlation with *H. suis* colonization rate (see additional files 6 and 8 for the r- and P-values).
